# Development of a High Min-Entropy Quantum Random Number Generator Based on Amplified Spontaneous Emission

**DOI:** 10.3390/e25050731

**Published:** 2023-04-28

**Authors:** Charlotte K. Duda, Kristina A. Meier, Raymond T. Newell

**Affiliations:** 1 Material Physics & Applications, Los Alamos National Lab, P.O. Box 1663, Los Alamos, NM 87545, USA; 2 Intelligence & Space Research, Los Alamos National Lab, P.O. Box 1663, Los Alamos, NM 87545, USA

**Keywords:** quantum random number generator, amplified spontaneous emission, min-entropy

## Abstract

We present the theory, architecture, and performance characteristics of a quantum random number generator (QRNG) which operates in a PCI express form factor-compatible plug-and-play design. The QRNG relies on a thermal light source (in this case, amplified spontaneous emission), which exhibits photon bunching according to the Bose–Einstein (BE) statistics. We demonstrate that 98.7% of the unprocessed random bit stream min-entropy is traceable to the BE (quantum) signal. The classical component is then removed using a non-reuse shift-XOR protocol, and the final random numbers are generated at a 200 Mbps rate and shown to pass the statistical randomness test suites FIPS 140-2, Alphabit, SmallCrush, DIEHARD, and Rabbit of the TestU01 library.

## 1. Introduction

Quantum mechanics is inherently non-deterministic [1], and thus a quantum system can serve as an ideal source to generate truly random numbers [2]. Early approaches to quantum random number generation were based on sources of radioactive decay [3,4] and shot noise in electronic circuits [5,6]. Later approaches used optical sources, which are easier to implement and include lasers [7], LEDs [8], vacuum noise [9], and single-photon sources [10]. Two important parameters used to quantify the performance of a random number generator are the bit generation rate and the entropy of the raw bit stream. Bit generation rates of around 100 Mbps [11,12,13] have been demonstrated using single-photon time measurement schemes, but the speed of single-quanta schemes is mainly limited by the count rate of single-photon detectors. In comparison, higher bit rates are possible for generators that employ conventional photodetectors (PDs). For example, schemes based on self-heterodyne detection of laser phase noise have reported bit rates of 68 Gbps [14]. Schemes based on the detection of amplified spontaneous emission (ASE) have also been proven to be promising, with bit rates of 16.8 Tbps recently being demonstrated [15]. In most cases, the bit stream produced will not be perfectly random and will require the use of a randomness extractor [16]. The use of such an extractor requires estimation of the entropy of the raw bit stream, which can be challenging, and if performed incorrectly, it can put the security of the scheme at risk. In addition, an extraction algorithm decreases the final bit rate and increases the complexity of the system.

We report on a quantum random number generator (QRNG) based on the detection of ASE using a conventional photodetector, which is high enough in entropy to not require the use of a randomness extractor [17]. Here, we detail one implementation of our QRNG where a semiconductor optical amplifier is used to generate ASE. Specifically, we use an optical amplifier where only one transverse field mode is amplified, leading to a large mean photon occupation number per mode. The probability of a photon being emitted into an already occupied field mode is then enhanced via a process called photon bunching, which is characteristic of bosonic particles [18]. This results in a large observed quantum signal-to-noise (QSN) ratio, where the random fluctuations in the photon occupation number are well above the shot noise.

In this paper, we present two implementations of our QRNG. First, a tabletop experiment is used to demonstrate that 0.987 bits of quantum entropy can be extracted per bit of raw output. The tabletop experiment is then simplified into a PCI express form factor-compatible plug-and-play design. The random bit stream generated using the PCI compatible QRNG is then shown to pass a comprehensive list of statistical tests, including FIPS 104-2, SmallCrush, Rabbit, Alphabit and Diehard of the TestU01 library [19], following shift-XOR conditioning [20].

## 2. Materials and Methods

**Figure 1:**
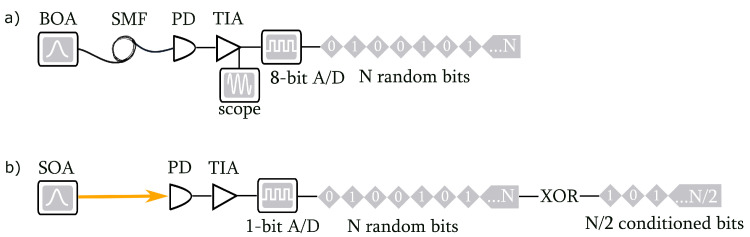
Two quantum random number generator implementations. (**a**) The tabletop experiment where BOA is the booster optical amplifier, SMF is a single-mode fiber, PD is the photodetector, TIA is the trans-impedance amplifier, and A/D is the analog-to-digital converter. (**b**) The PCIe-compatible experiment, where SOA is the semiconductor optical amplifier and XOR is shift-XOR conditioning.

The tabletop experiment is illustrated in Figure 1a. It consisted of a booster optical amplifier (BOA) centered at 1558 nm with a 10 THz bandwidth ($B_{op}$). The BOA output was fiber coupled to a 20 GHz bandwidth telecom-standard photodetector and amplified using a 600 MHz bandwidth trans-impedance amplifier. The analog signal was then characterized using an oscilloscope (MD04104-6, Tektronix, OR, USA) or digitized using an 8-bit A/D converter (NI PXIe-1082, National Instruments, TX, USA) operating at a sampling rate of 12 GHz with a threshold voltage of zero volts.

The PCIe-compatible experiment is shown in Figure 1b. The components had identical properties to the aforementioned tabletop experiment and were integrated into a board design. The primary difference was that the semiconductor optical amplifier (SOA) output was free space-coupled to the detector, and the number of sampled transverse field modes was controlled by the size of the detector’s active area. The analog signal was then digitized using a 1-bit A/D converter at the rate of 400 MHz.

The random-bit output of a length N was then streamwise shift-XOR conditioned using a non-reuse protocol implemented in a field-programmable gate array (FPGA). Two-bit streams *a* and *b*, both of a length N/2, were constructed by reading each byte from the raw bit stream in an alternating fashion. The autocorrelation value

(1) $$C\left(k\right)=\frac{\sum_{i=1}^{N/2}a_{i}⊕b_{i-k}}{N/2},$$

was then calculated, where stream *b* is shifted relative to stream *a* for values *k* = 0 to 127 bits. A bit-wise XOR operation is then performed, where a correlated result (0⊕0 or 1⊕1) is equal 1 and a non-correlated (0⊕1 or 1⊕0) is equal to –1. This process reduced the final conditioned bit generation rate to half the sampling rate of the A/D converter, which in this case was 200 Mbps.

## 3. Results

**Figure 2:**
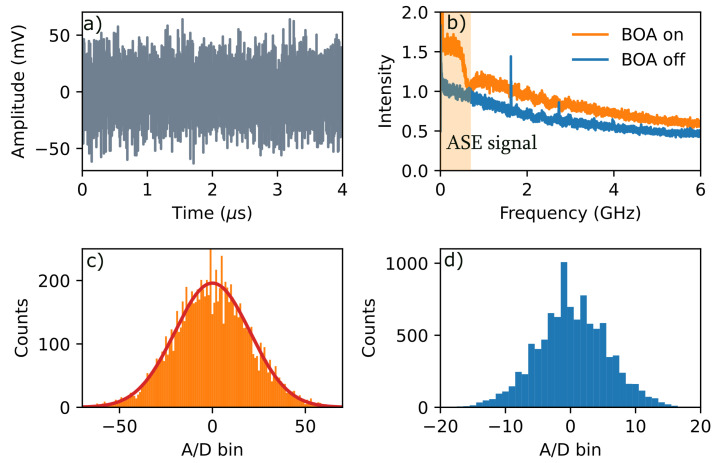
Characteristics of the tabletop QRNG averaging over 10 kbits, where BOA corresponds to a measurement at the maximum power received by the detector p=1.7 mW, which was achieved by running the BOA at the maximum drive current. (**a**) The measured time-domain trace on the oscilloscope operating at 3 GS/s. (**b**) The power spectrum measured after the A/D (analog-to-digital) converter. (**c**) Histogram of counts with A/D converter bin number for BOA switched on. (**d**) Histogram of counts for BOA switched off.

The power spectrum of the tabletop QRNG is shown in Figure 2b. A nominally 600-MHz bandwidth ASE signal centered at an arbitrary frequency is visible above the electronic noise floor. Figure 2c shows how the analog signal displayed in Figure 2a is digitized over the 256 available bins of the 8-bit A/D converter. The distribution is shown to be Gaussian, confirming that the random bits are equal instances of ones and zeros (i.e., the bit stream was unbiased). The min-entropy [21]

(2) $$H_{\infty }=-\log_{2}P_{max},$$

was evaluated by measuring the root mean square (RMS) amplitude from the time trace measurement at the oscilloscope, which is shown in Figure 2a. The contribution from the ASE signal to the measured value of the RMS power σ can be calculated using the expression for the photon count variance for an *M*-fold degenerate Bose–Einstein (BE) distribution [22,23]:

(3) $$\langle n^{2}\rangle =\langle n\rangle +\frac{{\langle n\rangle }^{2}}{M},$$

where 〈n〉 is the mean number of photons detected per 0.33 ns sampling bin, being equal to $4.37\times 10^{6}$ photons, and *M* is the number of longitudinal modes per sampling bin, being equal to $B_{op}/B_{el}=3.33\times 10^{3}$ modes [22], where the inverse of the sampling bin time sets the electronic bandwidth to $B_{el}=3$ GHz.

The first term in Equation (3) corresponds to the value of the shot noise photon count while the second term corresponds to the BE photon count. Therefore, the RMS BE power $\sigma_{BE}$ and RMS shot noise power $\sigma_{s}$ are equal to

(4) $$\begin{array}{c} \sigma_{BE}=\frac{\langle n\rangle }{\sqrt{M}}; \end{array}$$

(5) $$\begin{array}{c} \sigma_{s}=\sqrt{\langle n\rangle }. \end{array}$$

The outcomes with the highest probability can then be defined as

(6) $$\begin{array}{c} P_{max,\sigma }=\frac{1}{\sqrt{2\pi }\sigma }; \end{array}$$

(7) $$\begin{array}{c} P_{max,\sigma_{BE}}=\frac{1}{\sqrt{2\pi }\sigma_{BE}}. \end{array}$$

By substituting Equations (6) and (7) into Equation (2), we arrived at two expressions for the min-entropy of the system: one which represents the quantum min-entropy and the other which represents the min-entropy of the total signal. By taking the ratio of these two expressions, we arrived at a value of 98.7%, which represents the percentage of min-entropy associated with the quantum signal. This approach is justified by the properties of quantum mechanics, specifically that the quantum fluctuations are independent from all other sources of entropy.

**Figure 3:**
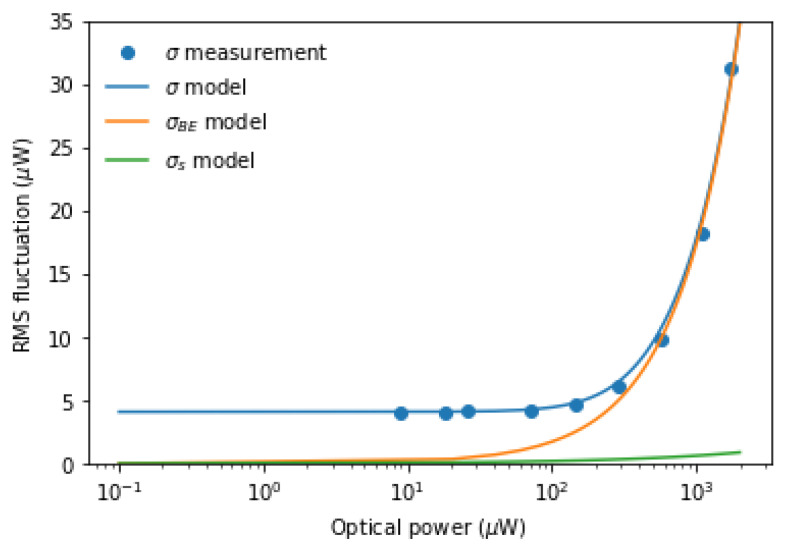
Measured root mean squared (RMS) power σ with optical power received by the detector *p*. Values were taken from the time trace at the oscilloscope of the tabletop experiment. The model curve σ was calculated using Equation (8), while $\sigma_{BE}$ and $\sigma_{s}$ were calculated using Equations (4) and (5), respectively.

Figure 3 shows the relationship between the measured RMS power σ and power received by the detector *p*. The model for σ was constructed by noting that σ will be equal to the root sum square of the RMS electronic noise power $\sigma_{e}$, $\sigma_{s}$, and $\sigma_{BE}$:

(8) $$\sigma =\sqrt{\sigma_{e}^{2}+\sigma_{s}^{2}+\sigma_{BE}^{2}},$$

In addition, the terms in Equation (8) will have the following power dependency:

(9) $$\begin{array}{c} \sigma_{e}=\sqrt{a}; \end{array}$$

(10) $$\begin{array}{c} \sigma_{s}=\sqrt{bp}; \end{array}$$

(11) $$\begin{array}{c} \sigma_{BE}=\sqrt{cp^{2}}, \end{array}$$

where a, b, and c are constants for a particular sampling bin time. Figure 3 shows $\sigma_{e}=4.09$ μW, while the model curves $\sigma_{s}$ and $\sigma_{BE}$ were constructed using Equations (4) and (5) to find $b=3.89\times 10^{-10}$ and $c=2.99\times 10^{-4}$, respectively. The model for σ is shown to agree well with the measurement over the complete range of optical powers. In addition, the contribution to σ is shown to be dominated by $\sigma_{BE}$, overwhelming the contributions from $\sigma_{e}$ and $\sigma_{s}$ at the maximum optical power p=1.7 mW. Specifically, at the maximum optical power, the quantum signal-to-noise ratio

(12) $$QSN=\frac{\sigma_{BE}}{\sqrt{\sigma_{e}^{2}+\sigma_{s}^{2}}},$$

was maximized at 7.06.

**Figure 4:**
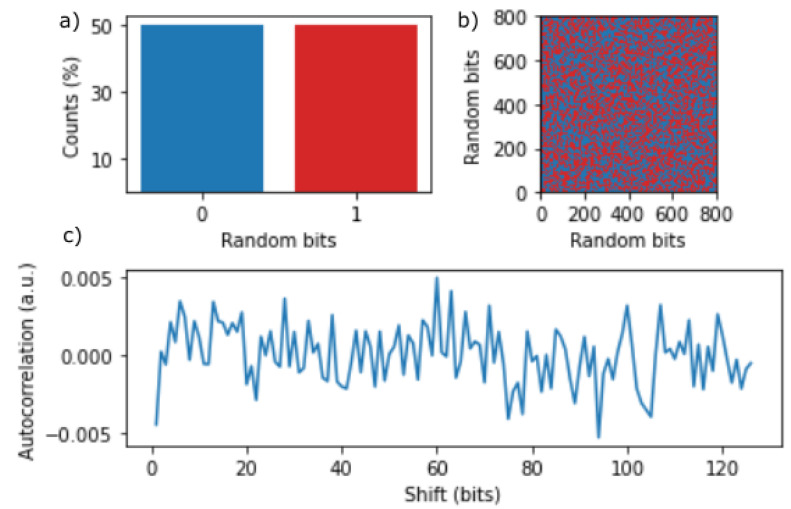
Statistics of the random bit stream generated from the PCIe-compatible QRNG for 80 kBytes. (**a**) The percentage counts for zero and one instances. (**b**) The bit pattern in a 2D plane, where zero and one are converted to blue and red dots, respectively. (**c**) The autocorrelation value C(k), calculated using Equation (1), with a shift value ranging from k = 0 to 127 bits.

The random bits generated using the PCIe-compatible QRNG (prior to non-reuse shift-XOR conditioning) are characterized in Figure 4. The percentage of zero and one instances is shown in Figure 4a, where the bias of the bit stream is confirmed to be close to zero with a 0/1 ratio of 319753/320247. Figure 4b shows the distribution of ones and zeros on a 2D plane, where no immediate pattern is discernible. Figure 4c shows the autocorrelation value (Equation (1)) as a function of the shift value using the shift-XOR non-reuse protocol outlined in Section 2.

A shift XOR of 24 bits was applied to create the conditioned random bit output of the PCIe-compatible QRNG. Both the unconditioned and conditioned random bits were then evaluated using the statistical randomness test suites of the TestU01 library: FIPS 140-2, SmallCrush, DIEHARD, Alphabit, and Rabbit. In this library, the battery FIPS 140-2 implements the small suite of tests from the FIPS 140-2 standard of the NIST library, and the battery DIEHARD applies most of the tests in the well-known DIEHARD suite of Marsaglia. Additional batteries (Alphabit, Rabbit, and SmallCrush), are also good tests of randomness. For the FIPS 140-2 standard with the SmallCrush and DIEHARD test suites, the data size evaluated in each test is predefined. For SmallCrush and DIEHARD, each test evaluates a new section of data, and 7.3 Gbits and 6.3 Gbits, respectively, were evaluated in total. For the FIPS 140-2 test suite, each test is evaluated on the first 20 kbits of data. For the Alphabit test suite, each test is evaluated over a different 100 Mbit section of data. Similarly, for the Rabbit test suite, each test evaluates a new section of data, where the maximum size is restricted to 100 Mbits. For all tests, a failed result is defined by a *p* value outside of the range of [0.001,0.9990].

**Figure A1:**
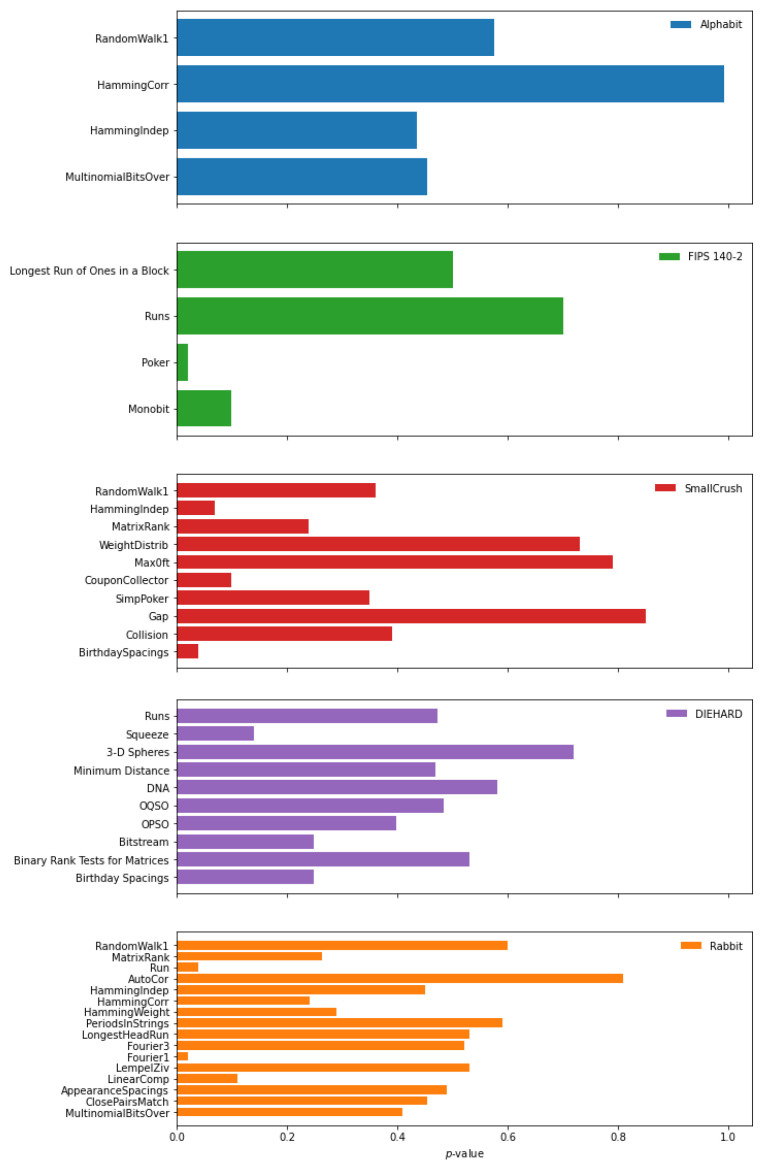
The recorded *p*-values for the statistical randomness test suites Alphabit, FIPS 140-2, SmallCrush, DIEHARD and Rabbit of the TestU01 library.

The unconditioned bit stream was shown to pass the FIPS 140-2 standard while failing numerous tests in the Alphabit, SmallCrush, DIEHARD, and Rabbit suites. In comparison, the conditioned bit stream was shown to pass all five test suites, with the recorded *p* values summarized in Figure A1. This result confirms that the non-reuse shift-XOR protocol is able to remove any short range correlations by using XOR for the bits separated in time. For example, short-range correlations may be introduced by the analog-to-digital converter, which has a finite analog bandwidth.

## 4. Discussion

We presented two implementations of an QRNG which is based on the transverse mode filtering of ASE to generate a high min-entropy bit stream. We demonstrated that 0.987 bits of quantum entropy can be extracted per raw bit of the output bit stream, which is a significant improvement in entropy quality when compared with presently available QRNGs. We then showed that the QRNG can be simplified and integrated into a PCIe plug-and-play design and pass the statistical randomness test suites of the FIPS 140-2 standard in Alphabit, SmallCrush, DIEHARD, and Rabbit of the TestU01 library, operating with a streaming rate of 200 Mbps following a small amount of shift-XOR conditioning.

Altogether, we demonstrated an QRNG which can be implemented using low-cost parts found in conventional telecommunication systems. In addition, the scheme also offers flexibility in design. For example, a super-luminescent diode would work equally well as a source, and an optical aperture could easily accomplish the mode filtering required for the scheme. Finally, our QRNG is also highly scalable, with the potential to operate with THz bit generation rates. In future implementations, one way to achieve this would be to use an arrayed waveguide grating to slice the ASE spectrum into multiple channels.

## 5. Patents

US patent 11442698B2.

## Data Availability

The datasets analyzed in this study are available at https://doi.org/10.17632/dw39sn74kg.1 (accessed on 4 April 2023).
